# Correction to ‘Modelling personality, plasticity and predictability in shelter dogs’

**DOI:** 10.1098/rsos.171590

**Published:** 2017-11-08

**Authors:** Conor Goold, Ruth C. Newberry

*R. Soc. open sci.* **4**, 170618. (Published Online 20 September 2017). (doi:10.1098/rsos.170618)

Lines 5–11 of the abstract should read as follows:

We modelled longitudinal observational data on shelter dog behaviour using the framework of behavioural reaction norms, partitioning variance into personality (i.e. inter-individual differences in average behaviour), plasticity (i.e. inter-individual differences in behavioural change) and predictability (i.e. individual differences in residual intra-individual variation).

